# Improved Method for Isolation of Neonatal Rat Cardiomyocytes with Increased Yield of C-Kit+ Cardiac Progenitor Cells

**DOI:** 10.4172/2157-7633.1000305

**Published:** 2015

**Authors:** Jennifer Rutering, Matthias Ilmer, Alejandro Recio, Michael Coleman, Jody Vykoukal, Eckhard Alt

**Affiliations:** 1 Department of Translational Molecular Pathology, Unit 2951, The University of Texas MD Anderson Cancer Center, 1515 Holcombe Boulevard, Houston, TX 77030, USA; 2 InGeneron Incorporated, 8205 El Rio Street, Houston, TX 77054, USA; 3 Applied Stem Cell Laboratory, Heart and Vascular Institute, Department of Medicine, Tulane University Health Science Center, 1430 Tulane Avenue, New Orleans, LA 70112, USA

**Keywords:** Cell isolation, Cardiomyocytes, Cardiac progenitor cells, Stem cells, c-Kit, Density gradient centrifugation

## Abstract

Cell therapy represents a promising new paradigm for treatment of heart disease, a major cause of death in the industrialized world. The recent discovery of tissue resident c-Kit+ cardiac progenitor cells (CPCs) has fueled scientific efforts to exploit these cells therapeutically for regenerative interventions, and primary culture of cardiomyocytes is a common *in-vitro* model to investigate basic molecular mechanisms underlying cardiac degeneration and regeneration. Current protocols for cardiomyocyte isolation frequently result in low cell yield and insufficient depletion of fibroblasts, which then overgrow the cardiomyocytes in culture. In this protocol we describe an improved method for the isolation of neonatal rat cardiomyocytes that also enables enhanced yields of CPCs. Gentle techniques of enzymatic and mechanical tissue processing ensure high cell numbers and viability, while subsequent Percoll density gradient centrifugation minimizes fibroblasts. We compared the advantages of different enzymes and found that Collagenase 2 alone leads to very high yields of cardiomyocytes, whereas the application of Matrase™ enzyme blend increases the relative yield of c-Kit+ CPCs to up to 35%. Cardiomyocytes and CPCs isolated with this protocol may constitute an important cell source for investigating heart disease as well as cell based therapeutic approaches.

## Introduction

Ischemic heart disease and heart failure remain among the leading causes of morbidity and mortality in the industrialized world [1]. The heart had been considered a post-mitotic organ without any regenerative capacity [2] fostered by the notion that cardiomyocytes withdraw from the cell cycle and terminally differentiate soon after birth [3,4]. However, this paradigmatic view has been altered considerably with numerous studies demonstrating the existence of cardiac-resident progenitor cells (CPCs) [5–7] that give rise to new cardiomyocytes and facilitate constant cellular turnover in the adult heart [8–10]. Since their initial discovery, various populations of CPCs have been characterized based on cardiosphere-formation [11] or expression of either receptor tyrosine kinase c-Kit (CD117) [12,13], stem cell antigen 1 (Sca1) [14] or Islet-1 [15]. A detailed gene mapping analysis recently showed different levels of cardiac commitment in each individual population, indicating that they may represent different states of the same progenitor cell [16]. Whereas Sca1+ and cardiosphere-derived cells (CDC) display a transcriptional profile closer to cardiomyocytes, c-Kit+ cells appear to be the most primitive and undifferentiated ones [17]. These c-Kit+ CPCs play a critical role in endogenous cardiac repair [18] and show the ability to reconstitute well-differentiated myocardium in an ischemic heart [12], which is fueling scientific interest to further explore their regenerative potential and applicability for therapeutic interventions. Cardiomyocyte cultures and c-Kit+ CPCs obtained by isolation from cardiac tissue serve as widely employed *in vitro* models. However, despite the fact that research on cardiomyocytes has been conducted for almost four decades [19], challenges remain regarding the primary isolation of these cells. Following enzymatic and mechanical dissociation of the heart tissue, a critical step of the isolation procedure lies in separating cardiomyocytes from non-contractile cardiac stromal cells such as fibroblasts, smooth muscle and endothelial cells. Fibroblasts rapidly proliferate and dominate these cultures, affecting cardiomyocyte phenotype and function [20,21]. Widely used commercially available cardiomyocyte isolation kits [22,23] do not efficiently address this issue of fibroblast separation, and the respective outcome of individual isolation protocols varies noticeably [24].

Regarding the isolation of CPCs, no standardized method has yet been established. Previous studies use regular protocols for enzymatic dissociation of heart tissue followed by sorting for the c-Kit+ cell population. The yields of c-Kit+ cells obtained with these methods, however, vary and can be quite low [5,13,25].

The objective of this study was to establish an improved protocol for primary cell isolation from cardiac tissue that ensures high yield, purity and viability of the isolated cardiomyocytes with specific enrichment of the c-Kit+ CPC population.

## Materials and Methods

### Tissue samples

Cardiac tissue was derived from the hearts of 1- to 2-day-old Sprague-Dawley rat pups. Animals were anesthetized with carbon dioxide and sacrificed by cervical dislocation. Hearts were removed and washed in ice-cold PBS (Invitrogen, Carlsbad, CA). Cardiac tissue was minced into pieces of approximately 1mm^3^ and washed again with cold PBS.

### Enzyme preparation

#### Matrase™ dissociation buffer

1 vial of Matrase™ enzyme blend (InGeneron Inc., Houston, TX) containing an average enzyme activity of 100 U was resuspended in 10 ml of cold sterile water. This enzyme solution was diluted up to 250 ml with cold sterile lactated Ringer’s resulting in an average activity concentration of 0.4 U/ml in the dissociation buffer.

#### Collagenase dissociation buffer

To obtain a 2% stock solution, 1 g of Collagenase 2 (Worthington Biochemical Corp., Lakewood, NJ) was dissolved in 50 ml of sterile lactated Ringer’s. 3 ml of this stock solution were diluted up to 100 ml with sterile lactated Ringer’s in order to achieve a final concentration of 0.12% (equivalent to 0.372 U/ml) in the dissociation buffer.

### Isolation of cardiomyocytes and CPCs

The choice of enzyme used for tissue processing was made depending on subsequent use of cells. We chose Collagenase dissociation buffer to obtain high numbers of cardiomyocytes, whereas Matrase™ dissociation buffer was used to maximize the specific yield of c-Kit+ cells.

Minced cardiac tissue was resuspended in respective enzyme buffer and processed for 15 minutes in the preheated ARC^®^ tissue processing unit (InGeneron Inc.). The enzyme buffer now containing isolated cells was recollected, transferred to a fresh tube and enzyme activity terminated by addition of cold horse serum. Fresh dissociation buffer was added to remaining tissue pieces and processing step repeated up to 9 times until tissue fragments were completely dissolved. Cell suspensions from all collecting tubes were pooled, centrifuged for 10 min at 350×*g* and the resulting cell pellet resuspended in cold ADS solution (ddH_2_O supplemented with NaCl, HEPES, NaH_2_PO_4_, Glucose, KCl, MgSO_4_, Phenol red).

### Percoll density gradient centrifugation

A two-layer density gradient was formed consisting of red-colored 63% Percoll solution underneath transparent 40.5% Percoll (GE-Healthcare, Uppsala, Sweden) solution. The cell suspension was layered on top of the gradient and tubes were centrifuged at 1,400 ×*g* and 4°C for 30 min using standard acceleration conditions and deceleration speed 0. Cardiomyocytes and CPCs could subsequently be removed from the newly formed layer between the Percoll solutions (band 2). Stromal cells including fibroblast equilibrate on top of the transparent Percoll solution (band 1) and were harvested separately. Collected material was pooled, diluted 1:4 in ADS and centrifuged at 620 ×*g* and 4°C for 5 min. The cell pellet was resuspended in cardiomyocyte growth medium (DMEM-F12 supplemented with 1% penicillin/streptomycin, 10% horse serum, NaHCO_3_, BSA, Sodium pyruvate, D-Glucose, Ascorbic acid, linoleic acid, transferrin, HEPES, Sodium selenite).

### Cell culture

Total number of isolated cells was counted and cell viability estimated via trypan blue staining. Cells were seeded at a density of 125,000 viable cells per cm^2^ on gelatin- coated multiwell-plates and placed in a humidified incubator at 37°C and 5% CO_2_. Cultures were left undisturbed for 24 hours and subsequent media changes performed every 48 hours. Movies of cultured cardiomyocytes were recorded utilizing a digital camera connected to a Zeiss Axiovert S100 microscope (Zeiss, Oberkochen, Germany).

### Flow cytometric analysis

Flow cytometric analysis was performed immediately after cell isolation as well as after 1, 4 and 7 days in culture. The antibodies used were anti α-actin monoclonal mouse IgM (Santa Cruz Biotechnologies, Santa Cruz, CA, cat. no. sc-58670), anti β-actin monoclonal mouse IgG1 (Invitrogen, Carlsbad, CA, cat. no. AM4302), AlexaFluor488-conjugated polyclonal donkey anti-mouse IgG (Invitrogen, cat. no. A-21202), DyLight594-conjugated polyclonal goat anti-mouse IgM (Abcam, Cambridge, MA, cat. no. ab97009) and FITC-conjugated monoclonal anti-CD117 mouse IgG (Millipore, Temecula, CA, cat. no. MAB1162F). Briefly, 5×10^5^ cells were collected and washed in flow buffer (PBS + 10% FBS + 1% Sodium azide). Direct extracellular staining of CD117 (c-Kit) was performed using the appropriate dilution of fluorescence conjugated primary antibody in flow buffer. Cells were then fixed in 100% methanol and incubated in permeabilization buffer (PBS + 0.5% Triton X-100). For intracellular indirect co-staining cells were stained with the primary antibody in blocking solution (PBS + 0.1% BSA + 0.2% Triton X-100 + 0.05% Tween-20 + 10% goat serum) and washed in PBS/T (PBS + 0.1% Triton) before incubated in the appropriate dilution of fluorescent secondary antibody. Analysis was performed by a FACSCalibur flow cytometer (BD Bioscience, San Diego, CA) and FlowJo software (Tree Star, Ashland, OR).

### Immunofluorescence staining

Immunofluorescence staining was performed after 4 days in culture using the following antibodies: anti α-actin monoclonal mouse IgM (Santa Cruz Biotechnologies, Santa Cruz, CA, cat. no. sc-58670), anti β-actin monoclonal mouse IgG1 (Invitrogen, Carlsbad, CA, cat. no. AM4302), AlexaFluor 488-conjugated polyclonal donkey anti-mouse IgG (Invitrogen, cat. no. A-21202) and DyLight 594-conjugated polyclonal goat anti-mouse IgM (Abcam, Cambridge, MA, cat. no. ab97009). Briefly, cells were fixed with 4% paraformaldehyde (Sigma-Aldrich, St. Louis, MO) and permeabilized (PBS + 0.5% Triton X-100). After incubation in blocking solution, intracellular staining was performed with the appropriate dilution of primary antibodies overnight. Then, cells were washed in immunofluorescence buffer (PBS + 0.1% BSA + 0.2% Triton X-100 + 0.05% Tween-20), and incubated in the appropriate dilution of respective fluorophore-conjugated secondary antibodies. Cell nuclei were counterstained with DAPI (Invitrogen) and subsequent analysis performed under an Axiovert S100 microscope using Nikon NIS Elements software (Nikon Instruments Inc., Tokyo, Japan).

### Data analysis

Analyses were conducted in three independent sets of experiments. The reported data are expressed as means ± SD. The statistical significance of the differences between groups was determined using the Student’s *t*-test. A level of p ≤ 0.05 was considered statistically significant, a level of p ≤ 0.01 was considered very significant.

## Results

### Work Flow

**Figure 1:**
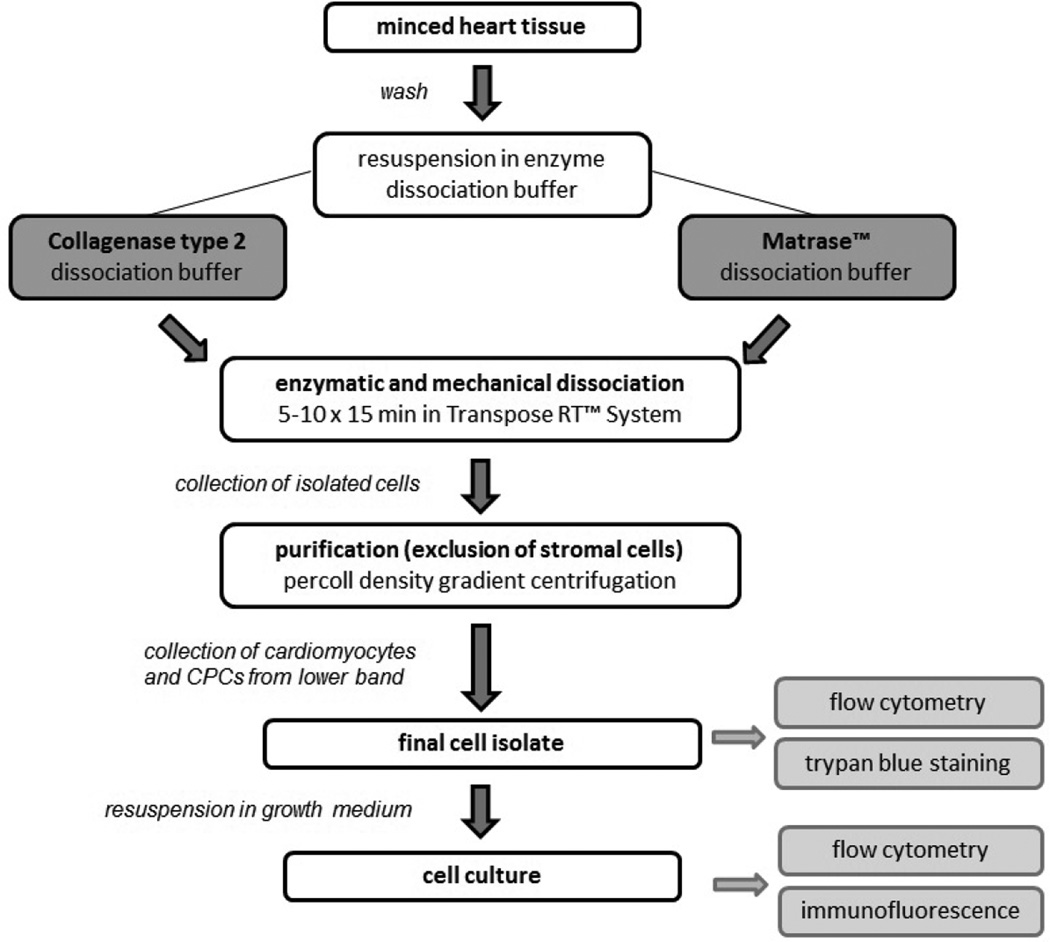
Work Flow of the isolation procedure.

Figure 1 illustrates the work flow of this protocol. The common procedure for cardiomyocyte isolation comprises enzymatic and mechanical dissociation of heart tissue followed by a purification step to reduce contamination by cardiac stromal cells. This protocol incorporates methods from previous studies [26–29] with several essential modifications. We developed two variations of the isolation procedure using either Collagenase 2 (Worthington Biochemical Corp.) or Matrase™ enzyme blend (InGeneron Inc.) for initial tissue dissociation. Incubation and mechanical agitation was carried out in the ARC™ tissue processing unit (InGeneron Inc.), a device specifically designed for primary cell isolation that provides optimal conditions for enzymatic tissue dissociation (Supplement Figure 1). Percoll density gradient centrifugation was subsequently employed. In order to objectively assess the advantages of this protocol, we compared it to the results obtained with a widely used commercially available isolation kit (Neonatal Rat Cardiomyocyte Isolation System, Worthington Biochemical Corp.) [22,23].

### Collagenase 2 and Matrase™ achieve higher viability and purity of isolated cells

**Figure 2:**
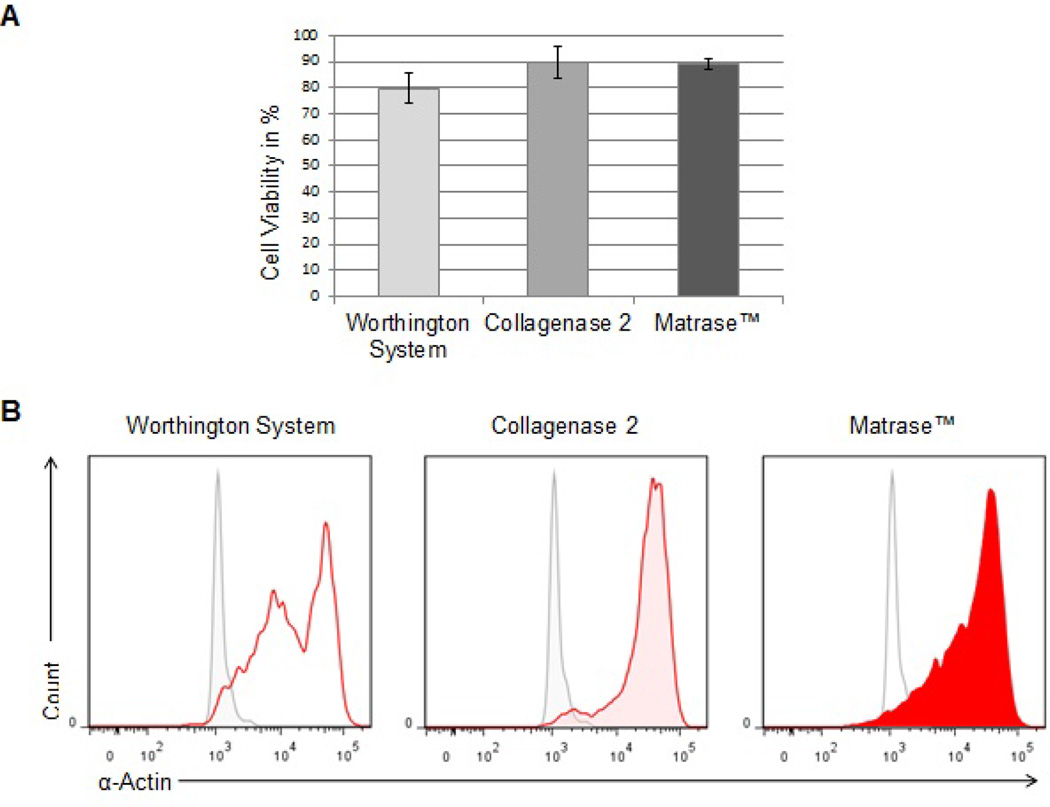
Purity and viability of the isolated cell population comparing different isolation methods. **(A)** Cell viability was assessed immediately after isolation by trypan blue staining. Analysis was performed in three independent sets of experiments. Data represent the percentage of viable cells relative to total cell number. **(B)** Freshly isolated cells were fixed, permeabilized and labeled with a DyLight 594 fluorescent antibody against cardiac-specific α-actin. Flow cytometric analysis was performed to compare the purity of the isolated cardiomyocyte population. The gray histogram represents the unlabeled control.

In three independent sets of experiments, three litters (equal to 36 hearts) of 1- to 2-day-old Sprague-Dawley rats were used for cell isolation yielding an average of 1.0 ± 0.2 × 10^6^ cells per heart. Trypan blue staining detected ~90% viable cells after dissociation with Collagenase 2 or Matrase™. In comparison, heart tissue that was processed with the Worthington Isolation System tended to yield only about 80% viable cells (p = 0.1719 and p = 0.0941) (Figure 2A). To assess the effectiveness of stromal cell separation, cells retrieved from Percoll density gradient centrifugation were immediately analyzed by flow cytometry for expression of α-actin. A-actin is a structural protein of myocytes and the antibody used for staining is recommended specifically for detection of cardiac muscle cells.

The Worthington isolation product revealed an inhomogeneous cell population with medium and high expression of α-actin; Collagenase 2 and Matrase™ predominantly yielded cells with high α-actin content indicating a markedly more pure isolation of cardiomyocytes (Figure 2B).

### Cell cultures of cardiomyocytes are homogenous and functionally intact

**Figure 3:**
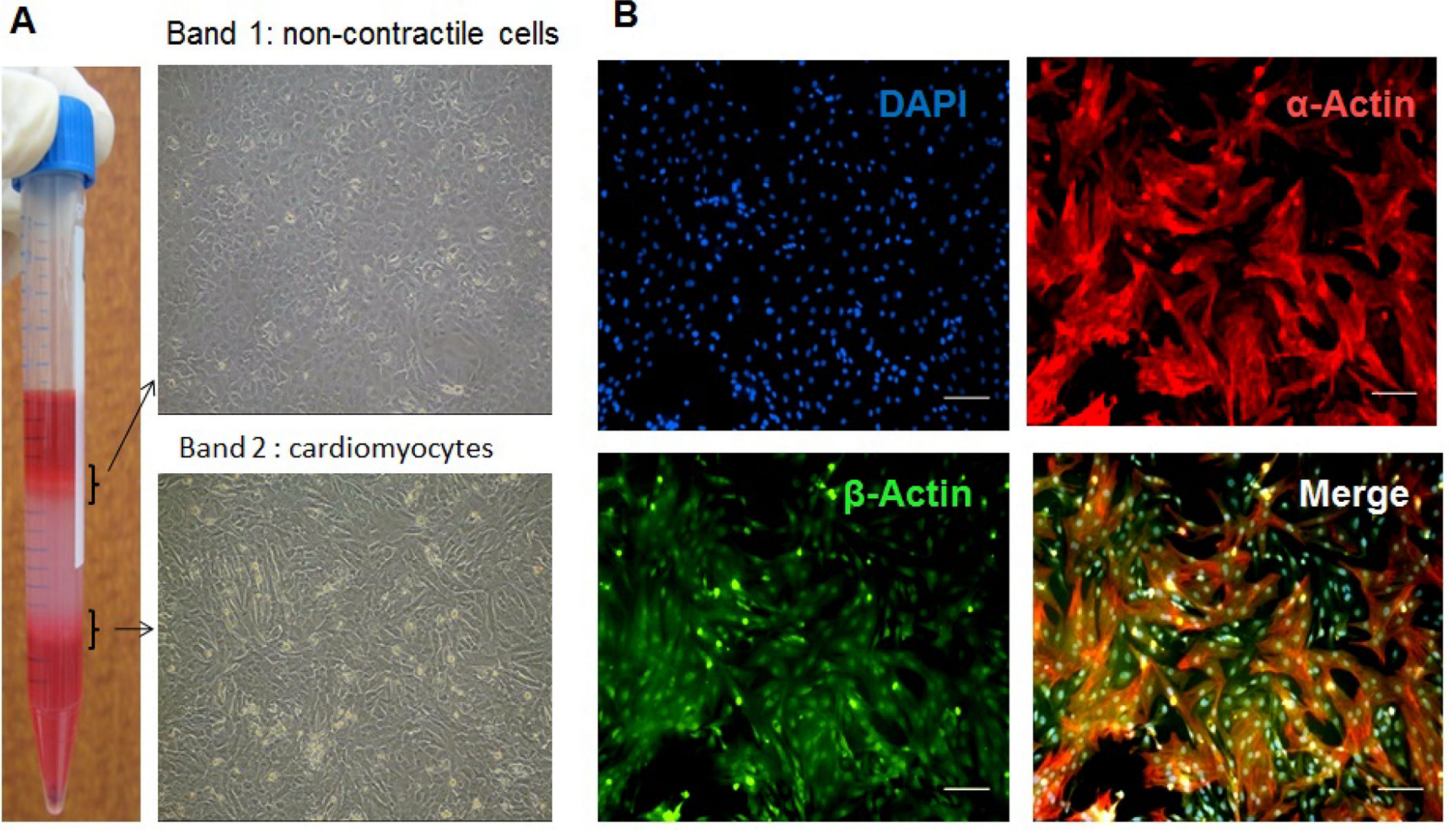
**(A)** Morphological characterization of cells retrieved after Percoll gradient centrifugation. Cells collected from newly formed bands 1 and 2 were cultured separately under cardiomyocyte growth conditions. Brightfield images taken after 4 days show a predominantly fibroblast-like morphology in the stromal cell fraction collected from band 1. Cells harvested from band 2 develop a typical rod-shaped cardiomyocyte phenotype and display rhythmic electric activity (corresponding videos can be found in the supplemental material (Suppl. Videos 2, 3)). **(B)** Purity of cardiomyocytes in culture. Cells were plated in 8-well-chamber slides and immunostaining was performed after 4 days in culture. Simultaneous expression of cardiomyocyte-specific α-actin (red) and ubiquitous marker β-actin (green) in almost all cells reveals a homogenous cardiomyocyte culture. Nuclei were counterstained with DAPI. Scale bar represents 100µm.

Percoll density gradient centrifugation usually leads to the typical appearance of a centrifuge tube (Figure 3A, left). Cells from the two newly formed layers (band 1 and band 2) between the Percoll solutions were harvested separately and cultured under regular cardiomyocyte growth conditions (see Methods). Morphological examination of the cultures reveals distinct cell phenotypes (Figure 3A, right). The cell population collected from band 1 predominantly contains spindle-shaped fibroblasts and shows no contraction ability (Supplement Video 1). Cells collected from band 2 display the typical rod-shaped cardiomyocyte morphology exhibiting spontaneous rhythmic contractions. They reach a confluence and electric synchrony after 2–4 days in culture (Supplement Video 2).

Immunofluorescence was performed after 4 days in culture. Co-labeling for the ubiquitous structural marker β-actin and cardiac specific-actin reveals simultaneous expression in almost all cells (Figure 3B). These findings indicate that stromal cells were successfully separated by Percoll density gradient centrifugation resulting in a homogenous and functionally intact cardiomyocyte culture.

### Isolation using Matrase™ yields high numbers of c-Kit+ cells

**Figure 4:**
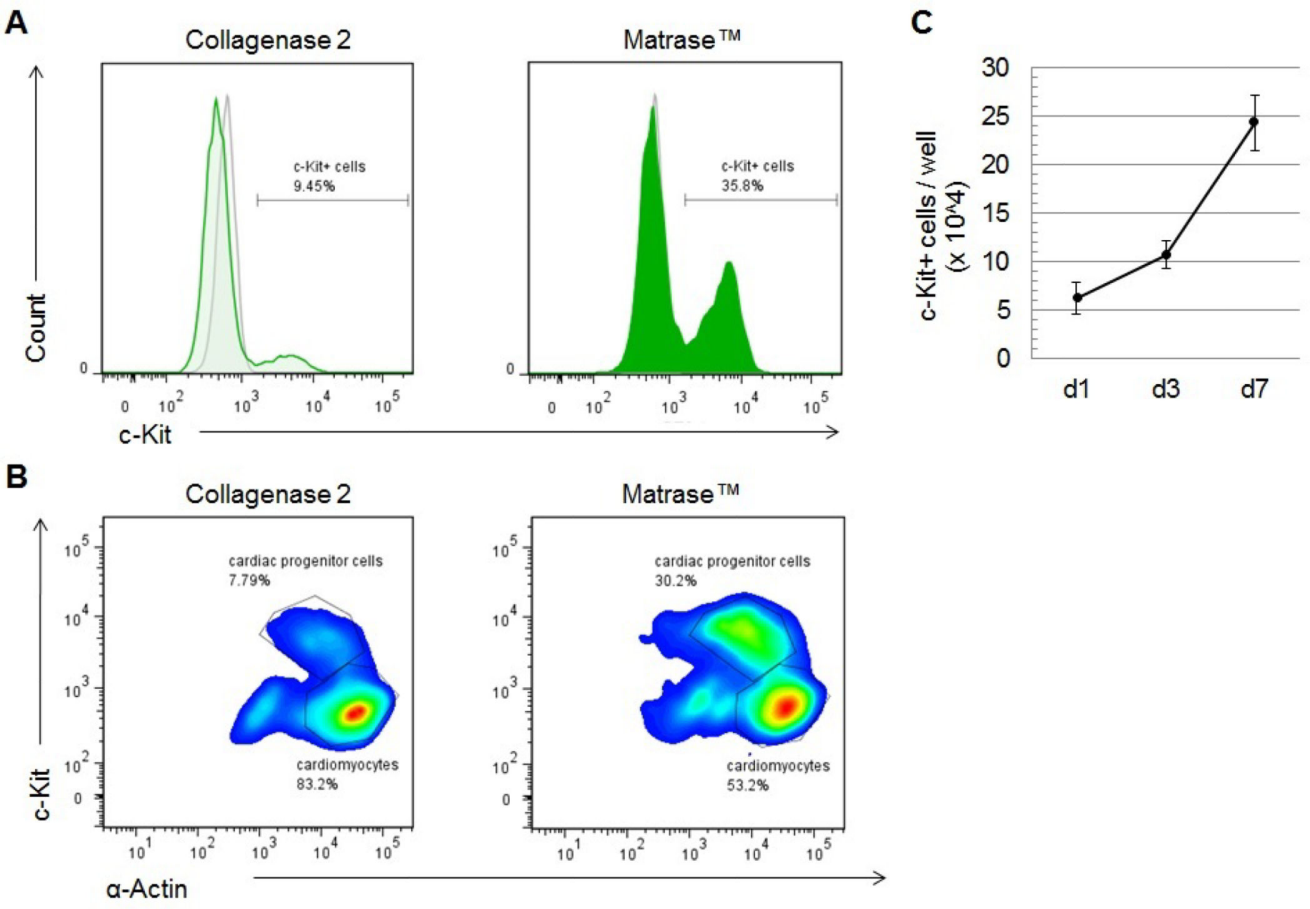
Quantitative analysis of c-Kit+ cells. **(A)** Freshly isolated cells were stained for c-Kit and α-actin and analyzed by flow cytometry. C-Kit-expression is more than 3-fold higher in the cell isolate after enzymatic dissociation using Matrase™. The gray histogram represents the unlabeled control. **(B)** Co-staining with α-actin reveals different populations within the cell isolate. C-Kit^low^ /α-actin^high^ cells are considered cardiomyocytes and represent the largest cell fraction in both isolation products. The population of c-Kit^high^ cells shows lower abundance of α-actin and is significantly higher after tissue processing with Matrase™. **(C)** In three sets of experiments, cells obtained from primary isolation were cultured in cardiomyocyte growth conditions and analyzed for c-Kit-expression after 1, 4 and 7 days. The constant increase of c-Kit+ cells indicates stable c-Kit- expression and maintenance of CPCs in culture.

The receptor tyrosine kinase c-Kit (CD117) is a widely accepted marker for cardiac progenitor cells (CPCs) [12,13,17,18]. In order to assess the yield of c-Kit+ cells obtained in both variations of this isolation protocol flow cytometric analysis was performed immediately after cell isolation. Representative results reveal the existence of two cell populations characterized by low versus high expression of c-Kit (Figure 4A). In the cell product obtained from isolation with Collagenase 2, cells with high expression of c-Kit account for 9.45% compared to 35.8 % in the isolate processed with Matrase™ (Figure 4A).

Co-staining for c-Kit and cardiomyocyte-specific α-actin facilitates further characterization of the isolated cells (Figure 4B). In both variations of the protocol, the dominant cell fraction is characterized by high levels of α-actin and low c-Kit- expression indicating that these are structurally mature cardiomyocytes. A distinct second population shows lower presence of a-actin, yet high expression of c-Kit, which allows the assumption that these represent c-Kit+ CPCs. After tissue dissociation using Collagenase 2 these c-Kit^high^/α-Actin^low^ cells account for 7.79% of the isolation product; tissue dissociation with Matrase™ led to a population of 30.2% c-Kit ^high^/α-Actin^low^ cells.

### Expression of c-Kit is maintained in culture

Cardiomyocytes and CPCs obtained from primary isolation were cultured under regular cardiomyocyte growth conditions (see Methods) and harvested at different time points (day 1, 3 and 7) for quantitative flow cytometric analysis. Results show that the number of c-Kit+ cells gradually increases in culture from 6.8 ± 1.62 × 10^4^ (day 1) to 10.7 × 10^4^ ± 1.45 (day 2, p = 0.0545) to 22.4 ± 2.87 × 10^4^ cells/well (day 7, p = 0.0039), which suggests persistent proliferative capacity and stable expression of c-Kit in the CPC population (Figure 4C).

## Discussion

This study introduces an improved method for the isolation of cardiomyocytes and c-Kit+ CPCs from neonatal rat hearts. The two variations of the protocol we present here differ only in the enzyme used for initial tissue dissociation and provide individual advantages. Our results indicate that the use of Collagenase 2 yields higher numbers of viable and pure cardiomyocytes, while enzymatic dissociation with Matrase™ remarkably increases the yield of c-Kit+ CPCs.

### Isolation of cardiomyocytes

An overview of existing neonatal cardiomyocyte isolation methods (Supplement Table 1) illustrates key advantages of this protocol as indicated by yield numbers, viability and purity of the final cell product. The total cardiomyocyte yield achieved in previously published methods ranges from 25,000 – 37,000 cells per heart or is not specified [24,27–31]. Viability of the isolated cells is between 70–90%, while purity of the cardiomyocyte population in most cases is not quantified. Using Collagenase 2 for tissue processing, we obtain a homogenous cardiomyocyte population with a viability of 85–90% and an average yield of more than 1 × 10^6^ cells per heart. These results can be attributed to several improvements regarding crucial steps of the isolation procedure. Most protocols employ trypsin in combination with collagenase for enzymatic tissue dissociation. We found that Collagenase 2 alone applied in sequential short time periods offers a more gentle way of processing the heart tissue (data not shown). This dissociation step was further optimized by using the ARC^®^ tissue processing unit instead of a conventional orbital shaker for mechanical agitation. The ARC^®^ tissue processing unit, which was specifically designed for primary cell isolation procedures, employs centrifugal agitation and temperature control to ensure optimal mixing and efficiency of enzymatic tissue dissociation. Several approaches have been suggested to minimize stromal cell contamination in cardiomyocyte cultures. The widely employed pre-plating technique [32], which separates cells based on different attachment properties, only removes 50–80% of non-cardiomyocytes [29], and the use of proliferation inhibitors such as Mitomycin C [23], Ara C [33] or BrdU [34] is limited due to the general cell toxicity of these components. Comparing different purification procedures, we found that density gradient centrifugation is the least harmful and most effective cell separation method (data not shown). We therefore optimized previously published methods using a discontinuous Percoll gradient [35] for application in our own protocol.

### Isolation of c-Kit+ CPCs

Since cardiac-resident c-Kit+ cells were first suggested as potential mediators of cardiac repair, interest and research in this field has rapidly expanded. Most previous studies perform conventional enzymatic dissociation of heart tissue and subsequently sort for the c-Kit+ cell population using either FACS (Fluorescence activated cell sorting) or MACS (magnetic activated cell sorting). The percentages of c-Kit+ cells obtained with these methods range from 0.7– 24% [5,12,13,25,36].

In the presented protocol using Matrase™, we yield numbers of up to 35% c-Kit+ cells among the cell isolate, obviating the need for further enrichment. This noticeable increase may be due to several major differences regarding experimental methods and conditions. First, the source of heart tissue used for cell isolation might have a significant influence on the anticipated yield of c-Kit+ cells. Whereas most studies utilize adult cardiac tissue [5,13,16,25] in this protocol cells were isolated from neonatal hearts, where the abundance of progenitor cells is suggested to be higher [36–38]. Furthermore, we have not removed the atria before tissue dissociation as often recommended in cardiomyocyte isolation protocols [24,29,39] since CPCs have been reported to accumulate especially in the right atrium [25,37]. Another essential difference is the insertion of a purification step before quantifying the separate cell populations. In most of the above-mentioned studies the percentage of c-Kit+ cells refers to the total number of cells isolated from cardiac tissue. The density gradient centrifugation implemented in this protocol *a priori* enriches for a particular cell population, in which the relative fraction of progenitor cells might therefore be reasonably higher.

Cardiac mast cells also express the c-Kit receptor. Although recent studies report that mast cell contamination is in fact relatively low after heart tissue dissociation [13,37], it should still be considered in the context of high numbers of c-Kit+ cells in the fresh cell isolate. Mast cells, however, usually are non-adherent and require very particular conditions for maintenance in culture [40]. The persistently high numbers of c-Kit+ cells observed in culture conditions as described here (Figure 4C) therefore argue against a significant contamination with mast cells in the primary cell isolate.

The 3-fold increase in the yield of c-Kit+ cells using Matrase™ versus Collagenase 2 in the otherwise identical isolation protocol can most likely be ascribed to the different enzyme properties. Matrase™ is a blend of highly purified and characterized recombinant enzyme preparations containing different types of collagenase as well as protease. Specifically developed for the dissociation of stem cells from connective tissue matrix [41], it could conceivably enhance the isolation of CPCs from their niches within the cardiac tissue. Compared to other studies that employ collagenase [5,13,16], trypsin [36] or dispase [25], this enzyme mix likely contributes to the noticeably higher yield of c-Kit+ cells in this protocol.

Regarding their promising regenerative potential, the question arises whether the isolated CPCs are viable under *in vivo* conditions. In previous studies using a similar isolation method (ARC^®^ tissue processing unit and Matrase™ enzyme blend), viability of cells from both animal [42] and human tissue [43] was found to be around 90%. In addition, it has been shown that these human-derived cells are able to survive and proliferate *in vivo* [44]. However, the applicability of the presented isolation method in human cardiac tissue as well as the viability of isolated CPCs under *in vivo* conditions are remaining questions that should be addressed in future studies.

### CPCs – hopes and perspective

Amongst the various stem and progenitor cells examined in this context, c-Kit+ CPCs have shown to be the most potent promoters of cardiac regeneration [45,46]. Based on successful application in animal models the first-in-man SCIPIO study was commenced in 2009 to investigate the effects of intracoronary autologous stem cell infusion in patients with post-myocardial infarction heart failure [47]. The encouraging initial results suggest a reduction in infarct size and improvement of left ventricular function after treatment with c-Kit+ CPCs. However, other reports have recently challenged the differentiation capacity of cardiac–resident c-Kit+ progenitor cells [48]. This fuels a controversial debate about benefits and limitations of stem cell therapy and shows that the understanding of cellular processes underlying cardiac repair is still limited [49]. Cardiomyocytes and CPCs isolated according this protocol may serve as an important cellular model for further investigating the role of progenitor cells in cardiac regeneration and pave the way towards stem cell based therapeutic approaches.

## Supplementary Material

Figure

material legends

table

video 2

video1
