# Supplementary figures and images for: Improved Method for Isolation of Neonatal Rat Cardiomyocytes with Increased Yield of C-Kit+ Cardiac Progenitor Cells
Source: J Stem Cell Res Ther. Author manuscript; Available in PMC 2016 Feb 29. (PMC4770583; doi:10.4172/2157-7633.1000305)

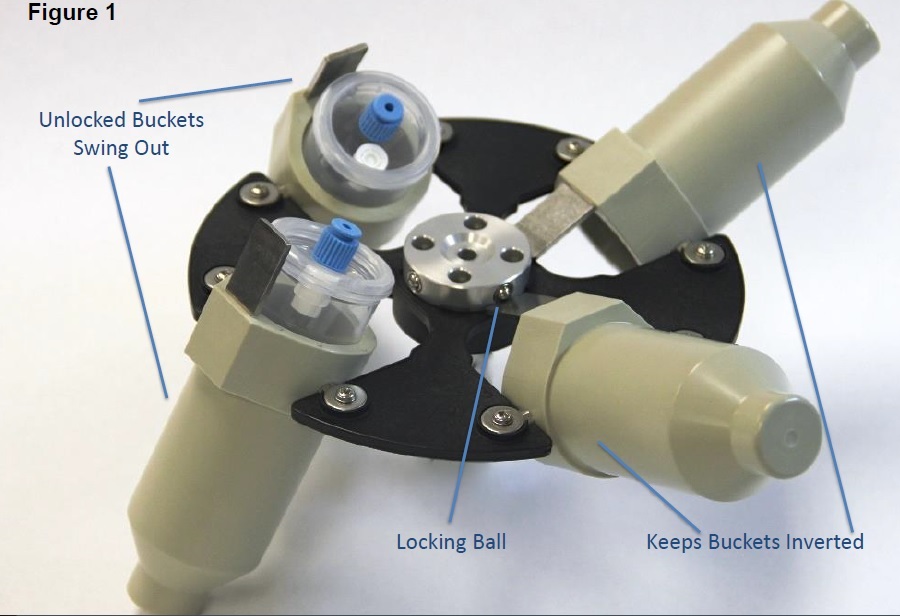

Supplement: Figure [file NIHMS755830-supplement-Figure.jpg]
