# Supplementary material for: Improved Method for Isolation of Neonatal Rat Cardiomyocytes with Increased Yield of C-Kit+ Cardiac Progenitor Cells
Source: J Stem Cell Res Ther. Author manuscript; Available in PMC 2016 Feb 29. (PMC4770583; doi:10.4172/2157-7633.1000305)
Supplement: material legends [file NIHMS755830-supplement-material_legends.docx]

**Supplemental Material Legends**

**Suppl. Fig. 1.** Picture of the rotor used in the ARC™ tissue processing unit. The flipbucket

has two different settings, a fixed and a swinging position. This enables for

both conventional centrifugation and mechanical agitation during tissue dissociation.

In this protocol we use the fixed position (right) with the inverted buckets. The

processing program performs sequential centrifugation intervals of a 5-sec spin

followed by a short stop, which ensures constant mixture and optimal enzymatic

dissociation of the tube contents.

**Suppl. Videos.** Morphology and function of separated cell fractions. Cells were

retrieved from band 1 and 2 (see Fig. 3A) after Percoll gradient centrifugation and

cultured separately under cardiomyocyte growth conditions. Brightfield videos were

recorded after 4 days in culture. **(1)** The cells collected from band 1 show a fibroblastlike

morphology and no contraction ability. **(2)** Cells from band 2 develop a typical

rod-shaped cardiomyocyte phenotype and form a monolayer exhibiting rhythmic

synchronous contractions.

**Suppl. Table 1.** Analysis of search results on PubMed for “Isolation of neonatal

cardiomyocytes”.
