# Supplementary material for: Improved Method for Isolation of Neonatal Rat Cardiomyocytes with Increased Yield of C-Kit+ Cardiac Progenitor Cells
Source: J Stem Cell Res Ther. Author manuscript; Available in PMC 2016 Feb 29. (PMC4770583; doi:10.4172/2157-7633.1000305)
Supplement: table [file NIHMS755830-supplement-table.doc]

| **Suppl. Table 1.** PubMed-analysis for "Isolation of neonatal cardiomyocytes" | | |  |  |  |
| --- | --- | --- | --- | --- | --- |
| **publication** | **comment** | **enzymatic dissociation** | **purification step** | **cell yield** | **viability** |
| **Methods in cardiomyocyte isolation, culture and gene transfer** | rather a review than a protocol | collagenase type 2 + pancreatin | percoll density gradient | - | - |
| Louch, William E.; Sheehan, Katherine A.; Wolskac, Beata M. |  |  |  |  |  |
| *J. Mol. Cell Cardiol., 2011* |  |  |  |  |  |
| **Neonatal rat cardiomyocytes--a model for the study of morphological, biochemical and electrophysiological characteristics of the heart** | review followed by a brief protocol | trypsin 0.2% (5 x 20 min) | pre-plating technique | - | - |
|  |  |  |  |  |  |
| Chlopcíková S, Psotová J, Miketová P. |  |  |  |  |  |
| *Biomed Pap Med Fac Univ Palacky Olomouc Czech Repub., 2001* |  |  |  |  |  |
| **Isolation and culture of neonatal mouse cardiomyocytes** | optimized for mouse cells | 1. isolation medium (overnight) 2. collagenase + dispase (20-30 min) | pre-plating technique | - | - |
| Ehler, E.; Moore-Morris, T.; Lange, St. |  |  |  |  |  |
| *Journal of visualized experiments, 2013* |  |  |  |  |  |
| **Production of spontaneously beating neonatal rat heart tissue for calcium and contractile studies** | focussing on cardiac stromal cells | trypsin + collagenase 2 + α-chymotrypsin (5-7 x 15 min) | percoll density gradient, collecting CM and FB as well as ILC (interlayer cells) | **CM:**  25,000 / heart | 70-90% |
|  |  |  |  | (purity not specified) |  |
| Gerilechaogetu, F.; Feng, H.; Golden, H.B.; Nizamutdinov, D.; Foster, D.M.; Glaser, S.; Dostal, D.E. |  |  |  | **ILC:** 37,500 / heart | 70-90% |
| *Cell-Cell interactions: methods and protocols, 2013* |  |  |  | **FB:** - | - |
| **Isolation of cardiac myocytes and fibroblasts from neonatal rat pups** | focussing on cardiomyocytes | trypsin + collagenase 2 + α-chymotrypsin (5-7 x 15min) | percoll density gradient, collecting CM | **CM:**  25,000 / heart | 70-90% |
| Golden, H.B.; Gollapudi, D.; Gerilechaogetu, F.; Li, J.; Cristales, R.J.; Peng, X.; Dostal, D.E. |  |  |  |  |  |
| *Cardiovascular Development, 2012* |  |  |  |  |  |
| **An improved protocol for primary culture of cardiomyocyte from neonatal mice** |  | trypsin-EDTA (0,05%) (3 x 5min) | pre-plating technique | - | 85-90% |
|  |  |  |  |  |  |
| Sreejit, P.; Kumar, S.; Verma, R.S. |  |  |  |  |  |
| *In Vitro Cellular & Developmental Biology - Animal, 2008* |  |  |  |  |  |
| **Improved method for isolation of neonatal cardiomyocytes with increased yield of c-Kit+ cardiac progenitor cells** | focussing on cardiomyocytes | Collagenase 2 (0,372 U/ml) (8-10 x 15 min) | percoll density gradient |  | 85-90% |
|  |  |  |  | **CM**: 1,300,000 / heart |  |
| Rütering, J.; Ilmer, M.; Recio, A.; Coleman, M.; Vykoukal, J.; Alt, E. |  |  |  |  |  |
|  | focussing on CPCs | Matrase™ (0.4 U/ml) (8-10 x 15 min) | percoll density gradient | **CM**: 400,000 / heart | 85-90% |
|  |  |  |  | **CPCs:**  35% |  |
| note: "-" indicates that information is not given in the publication |  | search performed on 12/05/2013 on www.pubmed.org | | | |
| abbr.: FB = Fibroblasts, CM = Cardiomyocytes, CPCs = cardiac progenitor cells |  | key words: "isolation, neonatal, cardiac myocytes, cardiomyocytes" | | | |
| IF = Immunofluorescence, FC = Flow cytometry |  | number of all results: 128 | | | |
